# Patient reported outcome measurements in chronic rhinosinusitis; assessing the correlation between RSOM-31 and VAS.

**DOI:** 10.1186/2045-7022-5-S4-P28

**Published:** 2015-06-26

**Authors:** Dirk Dietz de Loos, Lieke van der Meer, Wytske Fokkens

**Affiliations:** 1 Academic Medical Centre, Otorhinolaryngology, Amsterdam, Netherlands

## Background

Quality of Life (QoL) questionnaires are probably the most reliable outcome measurements to measure control of disease in CRS but can be relatively cumbersome. In certain situations a VAS (Visual Analogue Score) asking patients how their CRS is in general might be an easier evaluation tool.

## Aim

In this study we analyse the correlation between the RSOM-31 questionnaire, a VAS overall sinus’ score, and the comparable items measured as VAS.

## Methods

We collected RSOM-31, ‘VAS overall sinus’ score and VAS per symptom and analysed correlations.

## Results

705 CRS patients were included in this study (CRSwNP n=400). Correlation between mean RSOM-31 and ‘VAS overall sinus’ score showed a moderate correlation (r=0.52), as did the correlation between RSOM-31 nasal domain and ‘VAS overall sinus’ score (r=0.52). Excellent correlations between RSOM-31 and VAS were found for the comparable symptom specific questions; nasal congestion (r=0.80), rhinorrhea (r=0.83), sneezing (r=0.81), impaired sense of smell (r=0.82) and postnasal drip (r=0.86), all p-values <0.001. None of the individual RSOM-31 items correlated well with the VAS overall sinus’ score.

## Conclusion

We found a moderate correlation between mean RSOM-31 or RSOM-31 nasal domain and ‘VAS overall sinus’ score. The moderate correlation between these scores suggests patients, in both instruments, reflect different aspects of the burden of their disease. Further studies are needed to determine what patients include in their overall VAS score that is not captured with RSOM-31.

